# Current View on EpCAM Structural Biology

**DOI:** 10.3390/cells9061361

**Published:** 2020-05-31

**Authors:** Aljaž Gaber, Brigita Lenarčič, Miha Pavšič

**Affiliations:** 1 Department of Chemistry and Biochemistry, Faculty of Chemistry and Chemical Technology, University of Ljubljana, SI-1000 Ljubljana, Slovenia; aljaz.gaber@fkkt.uni-lj.si (A.G.); brigita.lenarcic@fkkt.uni-lj.si (B.L.); 2 Department of Biochemistry, Molecular and Structural Biology, Jožef Stefan Institute, SI-1000 Ljubljana, Slovenia

**Keywords:** **Keywords**: EpCAM, transmembrane protein, structure, dimer, adhesion, regulated intramembrane proteolysis, signaling, disease

## Abstract

EpCAM, a carcinoma cell-surface marker protein and a therapeutic target, has been primarily addressed as a cell adhesion molecule. With regard to recent discoveries of its role in signaling with implications in cell proliferation and differentiation, and findings contradicting a direct role in mediating adhesion contacts, we provide a comprehensive and updated overview on the available structural data on EpCAM and interpret it in the light of recent reports on its function. First, we describe the structure of extracellular part of EpCAM, both as a subunit and part of a cis-dimer which, according to several experimental observations, represents a biologically relevant oligomeric state. Next, we provide a thorough evaluation of reports on EpCAM as a homophilic cell adhesion molecule with a structure-based explanation why direct EpCAM participation in cell–cell contacts is highly unlikely. Finally, we review the signaling aspect of EpCAM with focus on accessibility of signaling-associated cleavage sites.

## 1. Introduction

EpCAM (CD326) is a conserved type I transmembrane glycoprotein of 35 kDa expressed on epithelial cells of higher eukaryotes. It was initially discovered as a carcinoma cell surface antigen [1] and has since then gained immense importance as a carcinoma marker with applications in diagnosis, prognosis, and therapeutic intervention [2,3]. Soon after EpCAM’s discovery it was proposed that it functions as a homophilic cell–cell adhesion molecule [4], hence its name—epithelial cell adhesion molecule. However, recent discoveries put this name to test [5,6]. Today, EpCAM implication in cell proliferation and differentiation is at the forefront, cell proliferation enhancing signaling via regulated intramembrane proteolysis [5,7], and regulation of epithelial-to-mesenchymal transition (EMT) [8,9,10]. The first high-resolution structure of EpCAM paved the way for these detailed studies [11] and here we review recent findings in the light of available structural data.

## 2. Evolution of the Structural Model of EpCAM

**Figure 1:**
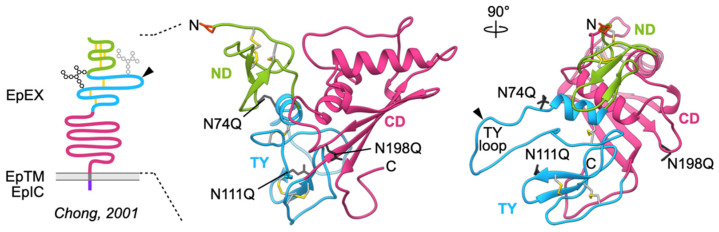
Structure of EpCAM extracellular part (EpEX). Left, adaptation of EpCAM model as presented by Chong et al. [15]; the N74 and N111 are shown as partially and fully glycosylated (gray and black, respectively), and the main protease-sensitive site marked by an arrowhead. Middle and right, crystal structure of EpEX (PDB 4MZV) in ribbon representation depicts the three domains (ND, TY and CD), disulfide linkages (yellow spheres), *N*-terminal pyroglutamate residue (orange-red sticks), and three glycosylation sites where asparagine was mutated to glutamine to abolish glycosylation and thereby achieve a homogenous protein sample for structure determination (mutations N74Q, N111Q, N198Q; dark gray sticks). Polypeptide chain is color-coded according to the domains and the same color coding is used throughout the paper. This and all other structural figures were prepared using UCSF Chimera version 1.14 (University of California San Francisco, Resource for Biocomputing, Visualization, and Informatics, San Francisco, USA) [16].

The first information on structural organization of EpCAM polypeptide chains was topology assignment deduced from the cDNA-derived protein sequence: a large extracellular part (EpEX), a single transmembrane region (EpTM or TM), and a short cytosolic tail (EpIC or IC). Within the extracellular part a thyroglobulin type-1A (TY) repeat [12] containing a protease-sensitive site [13,14] has been identified. The lack of other homologous and structural data resulted in erroneous classification of the *N*-terminal region between the signal peptide and the TY domain as an EGF-like repeat. This was finally addressed by disulfide linkage and glycosylation analysis [15] clearly identifying three domains within the extracellular part: a completely novel cysteine-rich *N*-terminal domain (ND), a TY domain, and a unique *C*-terminal domain with no cysteine residues (CD; Figure 1). These assignments were confirmed in 2014 by the crystal structure of the extracellular part of human EpCAM (Figure 1) which provided insight into EpCAM structure and function at several levels [11]. First, it revealed that the domains are not linearly arranged but rather form a triangular shape with the main protease-sensitive site Gly79–Arg80–Arg81 located on a loop protruding from the plane of the ND–TY–CD triangle. Next, the structure showed that the ND domain is structurally unique while the CD domain shares fold with some otherwise not related proteins.

**Figure 2:**
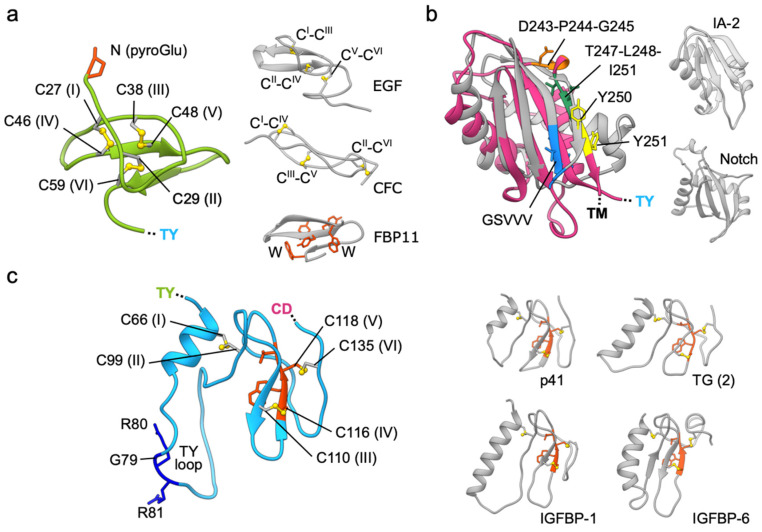
Domains of the EpCAM extracellular part. (**a**) ND (green ribbon) and its disulfide-packed core (yellow sticks); *N*-terminal pyroglutamate residue is shown in red sticks. Examples of other small cysteine-rich domains are shown on the right (gray ribbons): 9th EGF-like domain of human EGF (PDB 1JL9), CFC domain of murine Crypto (PDB ID 2J5H), and WW domain of human FBP11 (PDB 1YWJ) with characteristic tryptophan and tyrosine residues (orange-red sticks); disulfide bonds with connectivity order are shown as yellow sticks. (**b**) CD (deep pink ribbon) of EpCAM superimposed on the structure of the SEA domain of human MUC1 (gray; PDB 2ACM) which has an auto-proteolytic motif GSVVV (blue). Right, the sea urchin sperm (SEA) domains of human receptor-type tyrosine-protein phosphatase IA-2 (PDB 2QT7) and Notch receptor (PDB 3ETO), both without an auto-proteolytic activity, shown in the same orientation. Identified cleavage sites within CD by TACE (D243–P244–G245) and BACE (Y250–Y251) are shown in orange and yellow, respectively, and the AGR2-binding region is shown in dark green (overlapping the BACE cleavage site). The superposition was done using UCSF Chimera [16]. RMSD values range from 1.75 (IA-2 and EpCAM pair) to 3.31 Å (IA-2 and NOTCH1 pair) with an overall RMSD of 2.79 Å. (**c**) TY domain of EpCAM (left) with indicated disulfide bridges (yellow) and the protease-sensitive site GRR (dark blue). Homologous TY domains from p41 invariant chain (PDB 1ICF), thyroglobulin (TG, 2nd TY type-1 domain; PDB 6SCJ), and IFGBP-1 and -6 (PDB 1ZT3 and 1RMJ) are shown in gray. TY-characteristic CWCV sequence motif is shown in orange-red.

The *N*-terminal domain (just after the cleaved-off signal peptide, residues 24–63; ND) has a very compact core stabilized by a unique arrangement of three tightly packed disulfide bridges (Figure 2a) [11]. The *N*-terminal glutamine (Q24) is post-translationally modified to a pyroglutamate [15]. The domain’s orientation is stabilized via several polar as well as hydrophobic contacts with TY and CD. The disulfide bonding pattern is I–IV, II–VI, and III–V (Cys residues sequentially numbered from I to VI) which is clearly different from the canonical I–III, II–IV, and V–VI linkage found in EGF-like domains [17]. Likewise, the spacing between cysteine residues alongside the polypeptide chain differs between EGF-like domains and EpCAM ND. However, the ND does share disulfide linkage with some other small cysteine-rich domains, like the CFC domain of human cripto [18], and is with regard to fold [19] most similar to the disulfide-free WW-type domains [20] found in various proteins like formin-binding protein 3, dystrophin and NEDD, still, all these combined structural aspects are not found in any other protein of known structure, thereby classifying the structure of EpCAM *N*-terminal domain as unique. Domains of this type, also called cysteine-rich microdomains (size up to 40 aa residues), are often involved in protein–protein interactions, an example is a well-known EGF–EGFR interaction [21], and are also considered as therapeutic scaffolds with a very stable central core [22]. Interestingly, the ND of EpCAM is targeted by the vast majority of anti-EpCAM monoclonal antibodies (mAbs) raised up to now, perhaps due to its high exposure at the membrane-distal tip of the molecule, as discussed in the following section. In contrast, the anti-EpCAM mAbs targeting the TY or CD are rare [11,23].

Contrary to the ND, the C-terminal domain (residues 139–265; CD) is the largest of the three extracellular domains and contains no disulfide bridges. The domain has an α + β fold where the helices are clustered on one side of an antiparallel concave β-sheet (Figure 2b) [11]. While this domain does not have any significant sequence similarity to proteins outside of the GA733 family, the structural comparison revealed that it belongs to the SEA (sea urchin sperm protein, enterokinase, agrin) group of protein domains found in various cell surface and secreted proteins, for example in mucin MUC1, dystroglycan, receptor-type protein phosphatase IA-2 and Notch receptors (Figure 2b) [24]. The presence of this domain type in other distantly related eukaryotic lineages, for example in green algae, suggests an ancient evolutionary origin [24]. The function of these domains is not clear; however, some are implicated in signaling via proteolytic cleavage. The domain is often found paired with a transmembrane domain, as in EpCAM and all proteins listed here; in some proteins it possesses an auto-proteolytic activity through the GSφφφ motif (φ marks a residue with a hydrophobic side chain), for example in MUC1 [25]. While EpCAM CD lacks this auto-proteolytic motif, it is a subject of proteolysis by other proteases with implications in signaling [5] as discussed in detail later. Interestingly, one of these cleavage sites, the Y250–Y251, is juxtaposed to the spot corresponding to the auto-proteolytic site in MUC1 (Figure 2b). Contrary to other SEA domains, the CD of EpCAM contains a TLIYY motif (residues 247–251) which acts as a binding site for oncogenic ER-resident protein disulfide isomerase which may influence its trafficking [26].

The thyroglobulin-like domain (residues 64–138; TY) is sandwiched between the ND and CD. It is similar by sequence as well as by structure (Figure 2c) to TY domains of other functionally diverse vertebrate proteins: EpCAM paralogue Trop2, p41 invariant chain (TY as inhibitor of cysteine cathepsins), insulin growth factor-like binding proteins (IGBFPs; TY aids in binding IGF), SMOCs, testicans (TY as inhibitors as well as substrates of cysteine cathepsins), nidogens (TY as inhibitors of cysteine cathepsins), and thyroglobulin (some TY domains harbor precursor residues for thyroid hormones). These domains appeared early in the evolutionary history, however they are Metazoa-specific; in vertebrates the TY domain-containing proteins acquired other vertebrate-specific domains by exon shuffling and duplication [27,28]. On the basis of three disulfide bonds and bonding pattern I–II, III–IV, and V–VI, the TY domain of EpCAM is classified as TY type-1A domain (subtype 1B contains two disulfide bonds). Other characteristic features of the domain are a small hydrophobic core composed of *N*-terminal α-helix and β-sheet, stabilized by the three disulfide bridges, and the CWCV signature sequence motif (Figure 2c). The TY domains of various proteins differ by the length of loops and the *N*-terminal α-helix, and these structural differences are also linked to their functional diversity. For example, only TY domains with a short α-helix and short loops can act as inhibitors of cysteine cathepsins since longer loops sterically prevent inhibitor-style binding into the enzyme active site cleft [29]. EpCAM TY domain does not inhibit these or other peptidases, it is rather cleaved by them [11]—within TY domain is a dibasic protease sensitive site (G79–R80–R81) located at the protruding loop (Figure 2c). Matriptase, among other proteases, can efficiently cleave EpCAM at this site, thereby destabilizing its interaction with claudin-7 and, consequently, targeting it for internalization and degradation [30]. This protease-sensitive site is the same as the one identified and included in early models of EpCAM (Figure 1). The cleavage at this spot does not result in dissociation of the ND since parts of EpCAM remain tethered together via the C66–C99 disulfide bond within the TY domain (Figure 2c) [11].

## 3. The Biological Unit of EpCAM Is a cis-Dimer

**Figure 3:**
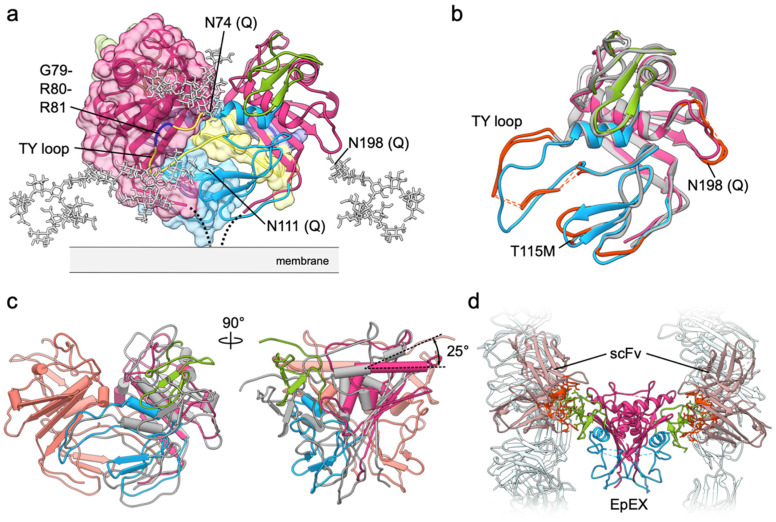
EpEX cis-dimer. (**a**) The EpEX cis-dimer is mostly stabilized by interactions between TY loop (yellow) and concave β-sheet of the CD. Both subunits are shown as ribbons, with one covered by molecular surface color-coded in the same manner. Modeled high-mannose glycans are shown as gray sticks. The protease-sensitive site within TY loop is shown in dark blue. (**b**) Superposition of one EpEX subunit from EpEX-only structure (PDB 4MZV; color-coded by domains) and of the two subunits from the EpEX-scFv structure (PDB 6I07; gray). Significant structural differences are marked with orange-red. Missing segments in the EpEX-scFv structure are shown as dotted lines. (**c**) Superposition of the cis-dimers (calculated over one subunit) from the EpEX-only and scFv-EpEX structures. For the superposed subunits only one is shown (salmon), for the other subunit the color coding is the same as in (b). (**d**) In EpEX-scFv structure (PDB 6I07) the scFv molecules (light gray-pink) interact via several residues (orange-red) with NDs of the EpEX dimer (color coded by domains, ND in green). Few other symmetry-related scFv molecules in the crystal are shown in light gray.

In the first crystal structure of EpEX two polypeptide chains from adjacent asymmetric units of the crystal form a crystallographic dimer [11]. Considering the extensiveness of interactions and large buried surface area upon dimer formation (1980 Å^2^ per subunit) with 8.6 kcal mol^−1^ solvation free energy gain it seemed likely that this dimer represents a biological unit. This was later confirmed by small angle X-ray scattering (SAXS) experiments in solution [6]. Moreover, structure revealed that EpCAM extracellular part alone is sufficient for dimerization, contrary to initial observations [31]. The dimerization interface is mostly formed by the protruding loop of the TY domain, which interacts with the concave β-sheet of the CD (Figure 3a). In such dimer the *C*-termini of the subunits are located close to each other, thus indicating that this arrangement represents a cis-dimer, in other words, formed by subunits from the same cell, since a trans-dimer (subunits from neighboring cells) would be impossible with such dimer architecture. Protein used for crystallization was a non-glycosylated mutant (three N-to-Q mutations; Figure 1), therefore *N*-glycosylation was modeled by attaching the canonical high-mannose chains to N74, N111 (both within TY) and N198 (within CD) (Figure 3a). While early reports indicated that N198 is not glycosylated, and the N111 and N74 are completely and partially glycosylated, respectively (Figure 1) [15], it was later shown that all three sites are indeed glycosylated and that abolishing glycosylation at N198 significantly and negatively affects overall expression level and half-life of EpCAM on the plasma membrane [32]. In the model of glycosylated cis-dimer the oligosaccharide chains protrude sideways and could be involved in maintaining proper orientation of EpCAM with regard to the membrane. This would contribute to a greater exposure of the glycosylation-free membrane distal part [11], especially of the ND. According to molecular dynamics simulations the dimer is additionally stabilized by dimerization of the transmembrane helices of the two subunits [11]. However, the dimerization interface most probably does not extend to the cytosolic tail (EpIC) considering the analogy to EpCAM paralogue Trop2—while it has been demonstrated that cytosolic tail of Trop2 has a strong potential to form α-helical structure, dimers were not detected [33]. For EpIC no stable or induced secondary structure has been observed (our unpublished results).

Additional insight into dimer architecture and dimerization-mediating interactions is provided by the second crystal structure of human EpEX in complex with a modified single-chain Fv fragment (scFv) from an anti-EpCAM Moc31 mAb (PDB 6I07) [34]. Similarly to the first structure, triple N-to-Q glycosylation-abolishing mutant protein was used, and the protein crystallized as a cis-dimer with two scFv fragments bound to each of the NDs, all within the same asymmetric unit, implying that the EpEX subunits are not related by a crystallographic axis and therefore not necessarily identical in structure. Few residues, mostly within the TY loop, were not resolved in the electron density map, however the overall subunit structure is similar with a RMSD over Cα atoms of 1.02 (Figure 3b) [34]. Some differences in the subunit structure are at the membrane distal part of the molecule where the EpEX-only structure accommodates a crystallization additive within a hydrophobic pocket causing a local conformational change, at a side-loop with the N198 glycosylation site (missing residues, or high B-factor indicating flexibility), in the TY loop, and near the CWCV motif of the TY domain. Here, in the EpEX-only structure the site 115 is occupied by a threonine residue while for the EpEX-scFv complex the methionine protein variant was used, resulting in a local structural change within this and the neighboring third loop of the TY without other far-reaching effects, since this region is not involved in extensive contacts with the rest of the subunit nor with the juxtaposed subunit. The T115M polymorphism has been linked to increased risk of breast cancer; however, the molecular mechanism is not known [35].

While in the two crystal structures the subunits are remarkably similar, there are substantial differences in their relative orientation as part of the cis-dimer (Figure 3c). Superposition of one subunit from each of the cis-dimer structures demonstrates that the other subunit in EpEX-scFv is inclined in comparison to the EpEX-only structure. This is clearly visible by the different relative angle of the membrane-distal α-helices which in EpEX-only structures run almost in parallel, while in EpEX-scFv structure they are at 25° angle. This poses a question, which of the two structures more closely resembles the biologically relevant cis-dimer? Due to missing residues (no electron density implying a local structural disorder) in the EpEX-scFv structure the direct comparison of interaction extensiveness would be inaccurate. However, since in the EpEX-only structure the TY loop critical for mediating cis-dimerization interactions is structurally well defined and forms extensive contacts with the CD (there is a well-defined electron density for the TY loop residues) [11], it is plausible that the EpEX-only structure (PDB 4MZV) indeed represents a more stable cis-dimer than the EpEX-scFv structure. Furthermore, crystal contacts can influence the cis-dimer structure. In the EpEX-only structure the crystal contacts are between the EpEX subunits/dimers themselves, while in the EpEX-scFv crystal they are mediated almost exclusively via scFv–scFv interactions between the adjacent asymmetric units and the EpEX dimers make little contact with the other neighboring EpEX dimers (Figure 3d). Effect of crystal packing on dimer conformation is a known phenomenon; an example is the Cro dimer from phage λ where subunits adopt different relative orientations in different crystal forms [36,37]. The fact that the two subunits could form a dimer with different relative orientations could indicate that the dimerization could be, although extensive in surface, partially dynamic. This would enable "breathing" of the dimer and might be associated with easier access to the proteolytic cleavage sites involved in signaling as discussed in the later section.

## 4. The Rise and Fall of EpCAM as an Adhesion Molecule from a Structural Point of View

EpCAM was first described as a calcium-independent cell–cell adhesion molecule, capable of mediating cell–cell adhesion through direct homophilic interaction between EpCAM molecules on adjacent cells [4,38]. The role in cell–cell adhesion was based on EpCAM translocation to the area of cell–cell contacts [38], disruption of cell–cell contacts by anti-EpCAM antibodies [38], and its ability to induce cell aggregation of mouse fibroblast cell line L929 lacking other endogenous adhesion molecules [4].

**Figure 4:**
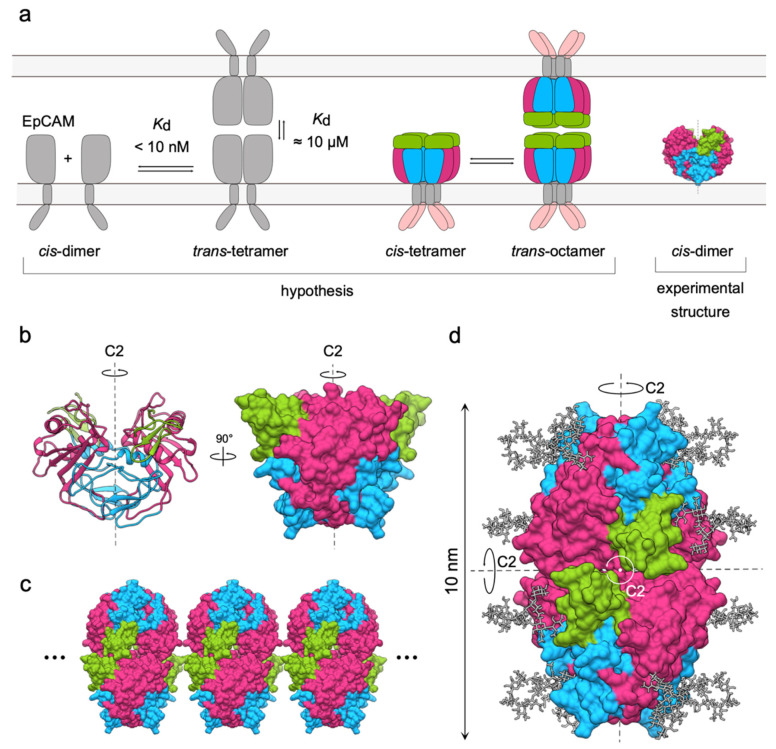
Models of EpCAM homo-oligomerization and formation of adhesion units. (**a**) The initial cis-dimer/trans-tetramer and cis-tetramer/trans-octamer hypotheses proposed in 2001 (left and center, respectively) and the experimental structure of EpCAM dimer from 2014 (right). (**b**) The symmetry of EpCAM cis-dimer in two different orientations, depicted as ribbons and molecular surface. Axis of rotation (C2) is shown as a dashed line. (**c**) Potential clustering of EpCAM adhesion units due to inherent symmetry implying lateral extension in both directions (dots). (**d**) Adaptation of the trans-tetramer model presented by Pavšič et al. in 2014 [11] with three C2 axes indicated by black dotted lines or a white dot.

Initially, there were two proposed models for the formation of EpCAM adhesion unit. The first one, based on chemical-crosslinking of full-length EpCAM (labeled as EpCAM from here on) and EpEX, and on analytical ultracentrifugation of EpCAM, suggested EpCAM forms trans-tetramers through interaction of two cis-dimers on opposing cells (Figure 4a, left) [31]. While the dimerization of full-length EpCAM was described as strong (dissociation constant; *K*_d_ < 10 nM), the trans-tetramerization appeared to be much weaker (*K*_d_ = 10 µM) [31], which is in agreement with initial observations that EpCAM-mediated adhesion is significantly weaker than adhesion mediated by E-cadherin [4]. Surprisingly, the same cross-linking experiments failed to detect any oligomerization of EpEX alone, suggesting that for oligomer formation full-length protein containing both the transmembrane and cytosolic parts is critical.

According to the second model EpCAM resides in the membrane as cis-tetramers that interact to form a trans-octameric cell–cell adhesion unit [39]. This mechanism was based on results of chemical-crosslinking and cell aggregation assays of truncated EpCAM, lacking one or more extracellular domains [39]. The TY domain was found necessary for lateral interactions, while the trans-interactions between proteins on opposing cells are mediated by ND (Figure 4a, center) [39].

A more detailed description of EpCAM adhesion unit was not possible until the high-resolution structure of EpEX was described in 2014 (Figure 1, Figure 3a and Figure 4a, right) [11]. This structure where EpEX was found in a cis-dimeric arrangement provided important insight into possible architectures of oligomers: the cis-dimer does not support the tetramer/octamer model of adhesion. EpCAM cis-dimer has a cyclic C2 symmetry with axis perpendicular to the cell membrane (Figure 4c). A lateral cis-tetramer, as proposed in the tetramer/octamer model, is thus unfeasible for two reasons. First, the interactions between the subunits in the cis-dimer need to be completely rearranged to enable formation of a different lateral homo-oligomer [40]. Considering the extensiveness of the cis-dimerization surface [11] the breakage of the cis-dimer would be prohibitive from the evolutionary perspective. Second, if C2 symmetry is not broken, a higher-order lateral homo-oligomer cannot have a finite number of subunits. Any interaction between two dimers that would lead to a formation of a tetramer would also imply an extension of the pattern via symmetry-equivalent sites resulting in tightly packed EpCAM clusters (Figure 4d). However, such clusters of EpCAM have never been observed [39,41].

In agreement with the dimer/tetramer model of adhesion, a D2-symmetric model of an EpCAM trans-tetramer was proposed [11] (Figure 4d). This model accounted for all up-to-date experimental knowledge on EpCAM adhesion and theoretical knowledge on oligomer evolution:

- The *C*-terminal of EpEX should extend towards the cell membrane as would be expected in the full-length EpCAM [11], thereby determining its basic orientation.
- The *N*-terminal domain is not relevant for cell–cell adhesion [39], suggesting that it is not directly involved in the adhesion-mediating interactions.
- The distance between cell membranes at sites of EpCAM mediated cell–cell contacts is around 10–14 nm [41]—this is roughly twice the dimension of an EpEX cis-dimer (5–6 nm) [11].
- *N*-glycosylation has no effect on adhesion [39] insinuating that sugar moieties do not participate in contact formation nor sterically hinder it.
- From an evolutionary point of view, dimers with cyclic C2 symmetry can evolve to form tetramers with dihedral D2 symmetry (three 2-fold axes of rotation, perpendicular to each other) [40] without disturbing the already existing symmetry.

However, all this experimental information was circumstantial—although this model was in agreement with all the above listed observations, there was no structural data available that could have been unambiguously attributed to the existence of EpCAM trans-tetramer. Moreover, there was also no evidence of higher-than-dimer order homo-oligomers when the crystal structure of the cis-dimer was determined [11]. Finally, no evidence of trans interactions between EpCAM cis-dimers was found in a comprehensive structural investigation of EpCAM oligomerization employing SAXS, cross-linking coupled with mass spectrometry (XL-MS), bead aggregation assays (BAA), and Förster resonance energy transfer (FLIM-FRET). SAXS showed that EpCAM extracellular parts in solution form stable cis-dimers in concentration range from 0.5 to 26.2 mg/mL (corresponding to 17.5–919.4 µM). Although chemical-crosslinking experiments were able to capture tetrameric EpCAM, the identified crosslinks between residues could be better explained by random and transient interactions of the cis-dimers in the solution rather than trans-tetramerization which would be, according to the adhesion model, biologically relevant. BAA, an experiment commonly employed to investigate cell–cell adhesion molecules, also showed no trans-interactions between EpCAM cis-dimers. Similar conclusions could be drawn from FLIM-FRET experiments, performed using two different cell lines. Combining these experimental observations, the authors concluded that EpCAM does not form higher-order homo-oligomers as has been assumed for more than 20 years [6].

Although this seemed to contradict all the previous experiments, it was not the first paper challenging the direct involvement of EpCAM in cell–cell adhesion via homo-oligomerization. First, Fornaro et al. failed to reproduce the initial results of EpCAM’s ability to induce cell segregation in transfected mouse fibroblast a year after the first such observations were described [42]. Next, Guillemot et al. also found that EpCAM has no effect on segregation of thymic epithelial cells [43]. Furthermore, Tsaktanis et al. published evidence that neither EpCAM cleavage nor EpCAM knockdown have any effect on cell–cell adhesion in a carcinoma cell line [5].

Combined, all the gathered experimental observations suggest that EpCAM’s role in cell–cell adhesion should not be attributed to homo-oligomerization of EpCAM molecules on opposing cells but rather to indirect regulation of classical E-cadherin mediated adhesion [44,45] and tight junction formation [46,47], actomyosin network homeostasis [48], and cell signaling.

## 5. Structural Basis of EpCAM Signaling via Regulated Intramembrane Proteolysis 

EpCAM is involved in several signaling pathways, and the first such evidence was provided in 2009 when regulated intramembrane proteolysis (RIP)-mediated signaling through EpIC–FHL2–β-catenin–Lef1 signaling complex was discovered [7]. Later it was also shown that EpCAM is involved in the MAPK signaling pathway through inhibition of nPKC [49,50], and that it regulates signaling through EGFR via direct binding to EGFR [9,51]. Most of the signaling pathways, except for RIP, have not yet been thoroughly investigated from the structural point of view.

**Figure 5:**
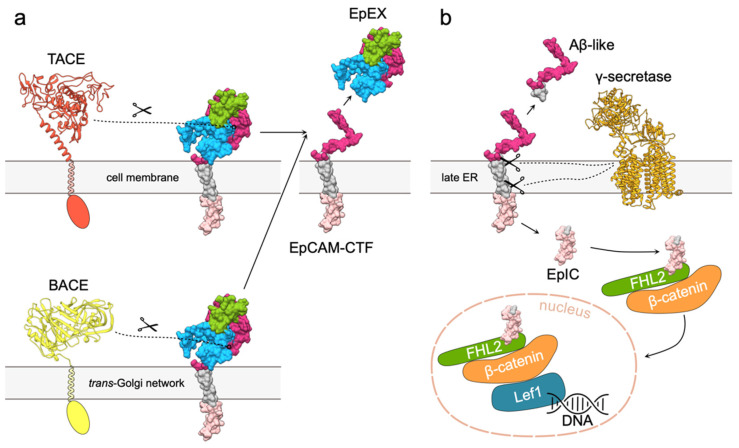
EpCAM signaling via RIP. (**a**) Cleavage of EpCAM extracellular part by either TACE or BACE results in release of soluble EpEX and membrane bound EpCAM-CTF. Extracellular part of TACE was modeled using Robetta [53,54], and structure extracellular part of BACE was obtained from the Protein Data Bank (PDB 2WJO). Transmembrane and cytosolic regions of both proteases are depicted schematically. EpCAM is presented as molecular surface; transmembrane region and intracellular domain are shown in gray and light pink, respectively. (**b**) Cleavage of EpCAM-CTF by γ -secretase complex (PDB 5A63) results in release of Aβ-like peptide and EpIC that is recruited in the EpIC–FHL2–β-catenin–Lef1 signaling complex. FHL (green), β-catenin (orange) and Lef1 (blue) are depicted by shapes corresponding to their relative sizes.

**Figure 6:**
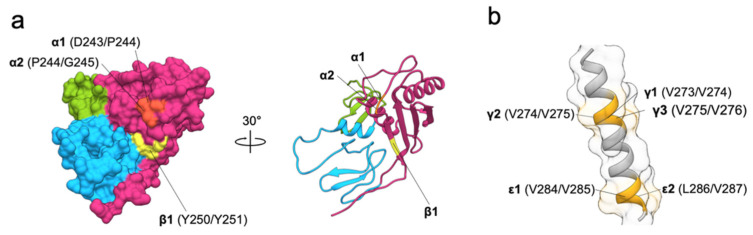
Structural details of RIP cleavages. (**a**) TACE (⍺1, ⍺2, orange) and BACE (β1, yellow) cleavage sites on EpEX shown in two different orientations. (**b**) γ-secretase (γ1–γ3 and ε1, ε2; light orange) cleavage sites in EpCAM transmembrane region.

In the case of EpCAM, RIP is comprised of two subsequent proteolytic cleavages. The first cleavage results in release of soluble EpEX and generation of a C-terminal fragment (EpCAM-CTF) (Figure 5a) [7,52]. It is mediated by either a disintegrin and metalloproteinase (ADAM) 17, also known as tumor necrosis factor-α-converting enzyme (TACE) [7], or β-secretase 1 (BACE) [52]. TACE and BACE cleavages, also termed α- and β-cleavages, occur at distinct locations. α-cleavage takes place at the plasma membrane, while β-cleavage is executed after EpCAM internalization, since BACE is predominately located to the trans-Golgi network. Cleavages at sites that are located at the C-terminal part of EpEX (Figure 6a) result in three possible soluble extracellular part variants [5]. However, it remains to be discovered if they differ in biological function. It is also not clear whether a single EpCAM molecule can be cleaved both at α- and β-sites and whether the type of cleavage influences downstream processing of EpCAM-CTFs.

The second cleavage (γ-cleavage) takes place in the ER where EpCAM-CTFs are processed by presenilin-2, a part of the γ-secretase complex (Figure 5b) [7]. γ-cleavage sites were pinpointed to five distinct positions within the EpCAM transmembrane region (Figure 6b), and the cleavage at first three (γ1–γ3) results in a soluble Aβ-like fragment that is released in the extracellular space. The last two (ε1, ε2) result in release of the soluble EpIC into the cytoplasm [5,52].

Although the name "Aβ-like" suggests a function similar to β-amyloid fragments, the biological function of EpCAM Aβ-like fragment is still to be discovered—the name only implies similar mechanisms of generation [52,55]. The role of EpIC, on the other hand, is much better understood. Soluble EpIC forms a complex with four-and-a-half LIM domain protein 2 (FHL2) and β-catenin that is in turn translocated to the nucleus where it interacts with transcription factor Lef1 [5,7] (Figure 5b). The resulting EpIC–FHL2–β-catenin–Lef1 signaling complex induces transcription of cell proliferation-related genes such as *CCNA2*, *CCND1* and *CCNE1* (cyclins A2, D1 and E, respectively), and *MYC* (c-myc) [7,56,57]. Recently, it has been discovered that generation of EpIC by γ-secretase is slow and that the resulting EpIC is afterwards efficiently degraded by the proteasome [58]. While this suggests EpIC is not suited for fast nuclear signaling as initially expected, it is still believed to be the main mechanism of EpCAM function as a signaling molecule.

**Figure 7:**
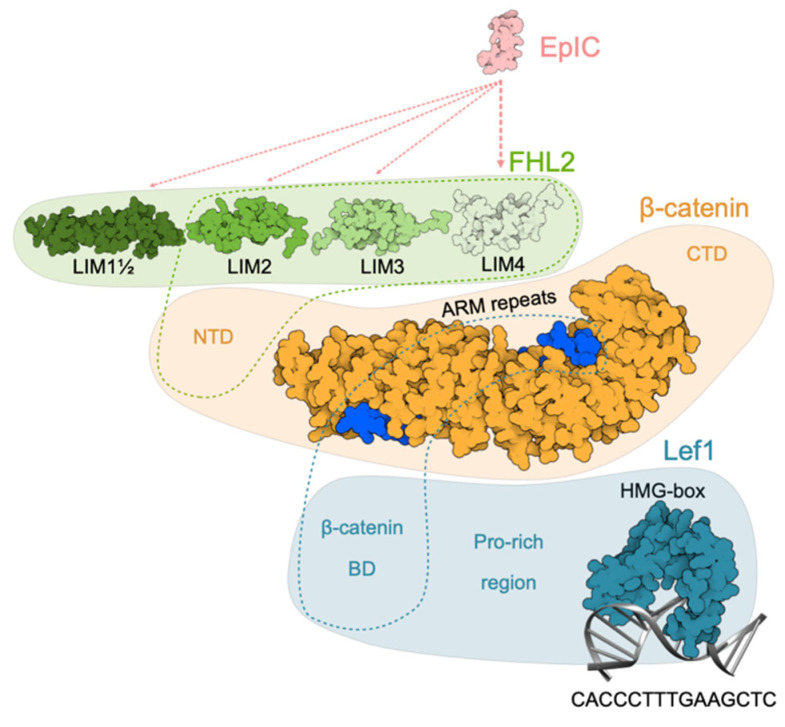
Schematic model of EpIC–FHL2–β-catenin–Lef1 signaling complex. EpIC was modeled using MODELLER [64]. Binding of EpIC to FHL2 is indicated by dotted lines (light pink; width is related to importance of interaction). First and a half, second, third and fourth domain of FHL2 are depicted based on corresponding NMR structures (PDB 2MIU, 1X4K, 2D8Z, and 1X4L respectively). Binding of FHL2 to β-catenin *N*-terminal domain is indicated by a green dotted outline. β-catenin is represented by structure of ARM repeats with bound part of Lef1 β-catenin BD (PDB 3OUW) and relative positions of *N*- and *C*-terminal domains (NTD and CTD, respectively), the structures of which are yet unknown. Position of β-catenin BD is indicated by blue dotted outline. Structure of Lef1, except for the *C*-terminal HMG-BOX bound to its target DNA sequence (PDB 2LEF), is not known. β-catenin BD and Pro-rich region are indicated at their relative position.

Structural information on the EpIC–FHL2–β-catenin–Lef1 signaling complex is sparse. The interacting pairs of proteins have been identified, but there is a lack of high-resolution structural data. However, some conclusions can also be drawn from structural investigations of β-catenin/Wnt-signaling pathway. First, EpIC interacts with FHL2 but not directly with β-catenin [59,60]. For interaction fourth LIM domain of FHL2 is crucial but the involvement of other LIM domains is not excluded [7]. Second, at minimum the last three LIM domains of FHL2 are needed for its interaction with β-catenin [61], but presence of the first and the half LIM domain increases the strength of the interaction. On the other hand, only *N*-terminal domain of β-catenin is needed for establishing a stable interaction [61]. The interaction between the full-length proteins is moderately strong (*K*_d_ ≈ 1.08 μM) [60]. Finally, crystal structure of β-catenin ARM repeats 2–10 with a bound part of Lef1 β-catenin-binding domain (β-catenin-BD) revealed that Lef1 interacts with β-catenin in an analogous manner as other members of TCF family. The affinities (dissociation constant) of β-catenin for Lef1 β-catenin-BD and its phosphorylated variant are 23 and 35 nM, respectively [62]. The structure of Lef1 HMG-box (291–391) bound to its target DNA segment was determined with NMR [63]. Considering all this structural data we build a schematic model of the complex (Figure 7).

**Figure 8:**
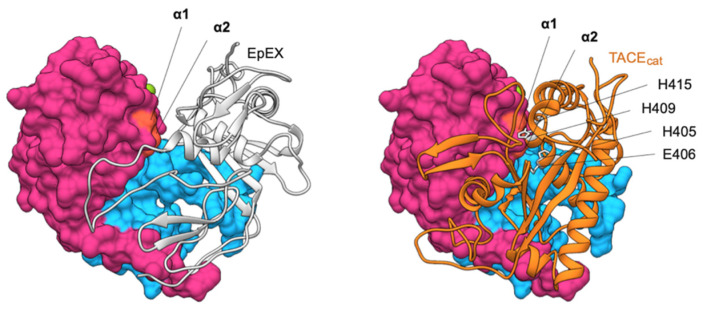
Comparison of EpEX cis-dimer structure (left) and model of EpEX–TACE_cat_ complex (right). One subunit of the dimer (gray ribbon) covers the cleavage site within the other subunit (molecular surface, cleavage site in orange). This cleavage site is in EpEX monomer easily accessible as shown by the complex of one subunit with a catalytic domain of TACE (TACE_cat_; orange ribbon). The model was generated using HADDOCK [67] with α1 and α2 cleavage sites on EpEX (orange surface) or TACE active and zinc binding site (H405, E406, H409, and H415; gray side chains) used as interaction restraints.

Despite considerable progress in our understanding of RIP-mediated EpCAM signaling in the past years several questions remain unanswered. First and most importantly, the exact role of EpIC in the EpIC–FHL2–β-catenin–Lef1 signaling complex is not known—β-catenin/Lef1 are known to induce transcription of the same oncogenes as EpIC-mediated signaling without the presence of either FHL2 or EpIC (reviewed in [65]). Second, the quest for identification of a RIP trigger has been, to date, unsuccessful. Initially it was proposed that soluble EpEX or formation of EpCAM cell–cell contacts initiates RIP, but this was later rebutted by discovering that such interactions are highly unlikely [6]. A recent report suggested that RIP is induced through EGFR activation via EGF [51] but others failed to confirm this observation [9]. Third, TACE cleavage sites were mapped on EpCAM cis-dimerization surface [5], meaning that cis-dimerization and cleavage are mutually exclusive (Figure 8). However, no explanation was provided on what causes otherwise stable EpCAM cis-dimers [6,11] to dissociate for the cleavage to take place. Similarly, EpCAM TM region has the tendency to dimerize [11] which may hinder its processing by γ-secretase as has been demonstrated for the *C*-terminal fragment of the amyloid β protein-precursor (APP CTFβ) [66].

Further investigation is needed to answer the abovementioned questions. We believe that such knowledge will not only provide us with a better understanding of this major EpCAM signaling pathway, but also pave the way for new possibilities for a rational design of the next-generation of drugs for treating carcinomas and other diseases involving EpCAM.

## 6. EpCAM Structure and Diseases

Since its discovery on the surface of colorectal cancer cells in 1979 [68,69], EpCAM has been recognized as an epithelial cancer antigen. Due to its frequent overexpression in carcinomas [70], it has been widely studied as a target for cancer diagnostics and treatment (reviewed in [71]). EpCAM overexpression is often linked to poor prognosis [72,73,74,75,76,77,78], presumably due to its involvement in cancer cell proliferation, migration, and metastasis [79]. 

Despite that, a lot of molecular details of EpCAM’s role in carcinogenesis are unknown. Surprisingly, there are not many known mutations in the EPCAM gene linked to cancer. Deletions of the last exons in one of the alleles are linked to hereditary non-polyposis colorectal cancer (HNPCC), also known as the Lynch syndrome [80,81,82]. In most cases these deletions involve loss of exons 8 and 9 that code for EpIC (for an extensive review please see [83]). Since in addition to other gene defects, the polyadenylation signal is also lost, it is not clear if these truncated forms are successfully translated. Hypothetically, such truncated proteins would not be able to participate in RIP-mediated signaling and their cell surface localization would be compromised due to disrupted localization-determining protein–protein or protein–lipid interactions. However, abnormal EpCAM protein is not believed to be the culprit at all—deletion of EpCAM 3’ exon causes silencing of a downstream DNA mismatch repair protein MSH2 gene through transcriptional read-through and promoter methylation [80], which results in increased risk of cancer. 

While monoallelic mutations are not known to cause developmental defects, biallelic mutations of the EPCAM gene cause congenital tufting enteropathy (CTE). CTE is an inherited disorder of the small intestine that results in a severe form of diarrhea [84]. To date, 42 different EPCAM mutations have been linked to CTE [83,85,86,87,88,89,90,91,92,93,94,95]. On the protein level these mutations result in single amino acid substitutions, truncations due to frameshifts, and missing segments due to abnormal splicing. In most cases EpCAM is synthesized as a soluble protein without its transmembrane and intracellular parts [83]. Mutant EpCAM (deletion of exon 4) is not present on the cell surface, it rather accumulates in the ER. This activates the ER stress-induced mechanism unfolded protein response (UPR) [96]. There are also two reports of homozygous full EPCAM gene knockouts [80,87]; however, they are lethal in mice [97].

Loss of functional EpCAM affects expression and proper localization of other proteins involved in cell–cell adhesion: key components of adherent junction E-cadherin and β-catenin [98], and tight junction protein claudin-7 [30,99,100]. This is easily explained when mutant EpCAM is expressed only as its soluble (truncated) extracellular part, because transmembrane and intracellular regions are responsible for interactions with claudin-7 [101] and β-catenin [7], respectively. However, the connection between structural and functional consequences of mutations is not immediately obvious in case of extracellular single amino acid substitutions, far from the identified interaction surfaces with the abovementioned proteins (for an extensive review please see [83]). We hypothesize that these mutations affect the stability of EpCAM protein as a whole and lead to either its increased internalization or proteolytic degradation or accumulation in ER, as in the case of exon 4 deletion mutant [96].

## 7. Concluding Remarks

Our understanding of EpCAM at structural level has improved significantly over the past decade. Determination of extracellular domain cis-dimeric crystal structure clarified its composition and domain organization. It also provided a detailed overview of EpCAM key structural features such as protruding *N*-terminal domain with a unique fold that harbors the majority of antibody binding sites, and a TY repeat with an uncommonly long loop that is critical for EpEX cis-dimerization via binding to its third, C-terminal domain, where important cleavage sites are located. The cis-dimer structure of EpEX also provided a basis for further investigation of the relationship between EpCAM homo-oligomerization and its role in cell–cell adhesion. However, recent findings provide compelling evidence that EpCAM molecules on the opposing cells do not interact, putting EpCAM’s adhesive role in question. In contrast, the number of studies reporting EpCAM engagement in signaling is increasing. Structural investigations of RIP cleavage explained the key steps in this process and provided an exact knowledge of cleavage sites. However, these findings brought about many new questions that need to be answered before all details of this signaling pathway are fully understood. For example, the relationship between cleavages and cis-oligomerization is not clear and the data on intracellular signaling complex is sparse.

More structural information on EpCAM interactions with its binding partners is also needed. We believe further endeavors in this direction will help us elucidate the complex and diverse role of EpCAM in epithelial morphogenesis, homeostasis, and disease.

## References

[1] Herlyn M., Steplewski Z., Herlyn D., Koprowski H. (1979). Colorectal carcinoma-specific antigen: Detection by means of monoclonal antibodies. Proc. Natl. Acad. Sci. USA.

[2] Keller L., Werner S., Pantel K. (2019). Biology and clinical relevance of EpCAM. Cell Stress.

[3] Murakami N., Mori T., Nakamura S., Yoshimoto S., Honma Y., Ueno T., Kobayashi K., Kashihara T., Takahashi K., Inaba K. (2019). Prognostic value of the expression of epithelial cell adhesion molecules in head and neck squamous cell carcinoma treated by definitive radiotherapy. J. Radiat. Res..

[4] Litvinov S.V., Velders M.P., A Bakker H., Fleuren G.J., O Warnaar S. (1994). Ep-CAM: A human epithelial antigen is a homophilic cell-cell adhesion molecule. J. Cell Boil..

[5] Tsaktanis T., Kremling H., Pavšič M., Von Stackelberg R., Mack B., Fukumori A., Steiner H., Vielmuth F., Spindler V., Huang Z. (2015). Cleavage and Cell Adhesion Properties of Human Epithelial Cell Adhesion Molecule (HEPCAM)*. J. Boil. Chem..

[6] Gaber A., Kim S.J., Kaake R.M., Benčina M., Krogan N., Šali A., Pavšič M., Lenarčič B. (2018). EpCAM homo-oligomerization is not the basis for its role in cell-cell adhesion. Sci. Rep..

[7] Maetzel R., Denzel S., Mack B., Canis M., Went P., Benk M., Kieu C., Papior P., Baeuerle P.A., Münz M. (2009). Nuclear signalling by tumour-associated antigen EpCAM. Nat. Cell Biol..

[8] Lin C.-W., Liao M.-Y., Lin W.-W., Wang Y.-P., Lu T.-Y., Wu H.-C. (2012). Epithelial Cell Adhesion Molecule Regulates Tumor Initiation and Tumorigenesis via Activating Reprogramming Factors and Epithelial-Mesenchymal Transition Gene Expression in Colon Cancer*. J. Boil. Chem..

[9] Pan M., Schinke H., Luxenburger E., Kranz G., Shakhtour J., Libl D., Huang Y., Gaber A., Pavšič M., Lenarčič B. (2018). EpCAM ectodomain EpEX is a ligand of EGFR that counteracts EGF-mediated epithelial-mesenchymal transition through modulation of phospho-ERK1/2 in head and neck cancers. PLoS Boil..

[10] Wang M.-H., Sun R., Zhou X.-M., Zhang M.-Y., Lu J.-B., Yang Y., Zeng L.-S., Yang X.-Z., Shi L., Xiao R.-W. (2018). Epithelial cell adhesion molecule overexpression regulates epithelial-mesenchymal transition, stemness and metastasis of nasopharyngeal carcinoma cells via the PTEN/AKT/mTOR pathway. Cell Death Dis..

[11] Pavšič M., Gunčar G., Djinović-Carugo K., Lenarčič B. (2014). Crystal structure and its bearing towards an understanding of key biological functions of EpCAM. Nat. Commun..

[12] Linnenbach A.J., Wojcierowski J., Wu S.A., Pyrc J.J., Ross A.H., Dietzschold B., Speicher D., Koprowski H. (1989). Sequence investigation of the major gastrointestinal tumor-associated antigen gene family, GA. Proc. Natl. Acad. Sci. USA.

[13] Thampoe I.J., Ng J.S., Lloyd K.O. (1988). Biochemical analysis of a human epithelial surface antigen: Differential cell expression and processing. Arch. Biochem. Biophys..

[14] Schön M.P., Schön M., Mattes M.J., Stein R., Weber L., Alberti S., Klein C.E. (1993). Biochemical and immunological characterization of the human carcinoma-associated antigen MH 99/KS 1/4. Int. J. Cancer.

[15] Chong J.M., Speicher D.W. (2000). Determination of Disulfide Bond Assignments andN-Glycosylation Sites of the Human Gastrointestinal Carcinoma Antigen GA733-2 (CO17-1A, EGP, KS1-4, KSA, and Ep-CAM). J. Boil. Chem..

[16] Pettersen E.F., Goddard T.D., Huang C.C., Couch G.S., Greenblatt D.M., Meng E.C., Ferrin T.E. (2004). UCSF Chimera–a visualization system for exploratory research and analysis. J. Comput. Chem..

[17] Wouters M., Rigoutsos I., Chu C.K., Feng L.L., Sparrow D.B., Dunwoodie S.L. (2005). Evolution of distinct EGF domains with specific functions. Protein Sci..

[18] Foley S.F., Van Vlijmen H.W.T., Boynton R.E., Adkins H.B., Cheung A.E., Singh J., Sanicola M., Young C.N., Wen D. (2003). The CRIPTO/FRL-1/CRYPTIC (CFC) domain of human Cripto. JBIC J. Boil. Inorg. Chem..

[19] Krissinel E., Henrick K. (2004). Secondary-structure matching (SSM), a new tool for fast protein structure alignment in three dimensions. Acta Crystallogr. Sect. D Boil. Crystallogr..

[20] Sudol M., McDonald C.B., Farooq A. (2012). Molecular insights into the WW domain of the Golabi-Ito-Hall syndrome protein PQBP. FEBS Lett..

[21] Ogiso H., Ishitani R., Nureki O., Fukai S., Yamanaka M., Kim J.-H., Saito K., Sakamoto A., Inoue M., Shirouzu M. (2002). Crystal Structure of the Complex of Human Epidermal Growth Factor and Receptor Extracellular Domains. Cell.

[22] Tombling B.J., Wang C.K., Craik D.J. (2020). EGF-like and Other Disulfide-rich Microdomains as Therapeutic Scaffolds. Angew. Chem. Int. Ed..

[23] Winter M.J., Nagtegaal I., Van Krieken J.H.J.M., Litvinov S.V. (2003). The Epithelial Cell Adhesion Molecule (Ep-CAM) as a Morphoregulatory Molecule Is a Tool in Surgical Pathology. Am. J. Pathol..

[24] Pei J., Grishin N.V.N.V. (2017). Expansion of divergent SEA domains in cell surface proteins and nucleoporin. Protein Sci..

[25] Levitin F., Stern O., Weiss M., Gil-Henn C., Ziv R., Prokocimer Z., Smorodinsky N.I., Rubinstein D.B., Wreschner D.H. (2005). The MUC1 SEA Module Is a Self-cleaving Domain. J. Boil. Chem..

[26] Mohtar A., Hernychova L., O’Neill J.R., Lawrence M.L., Murray E., Vojtesek B., Hupp T.R. (2018). The Sequence-specific Peptide-binding Activity of the Protein Sulfide Isomerase AGR2 Directs Its Stable Binding to the Oncogenic Receptor EpCAM. Mol. Cell. Proteom..

[27] Molina F., Bouanani M., Pau B., Granier C. (1996). Characterization of the Type-1 Repeat from Thyroglobulin, a Cysteine-Rich Module Found in Proteins from Different Families. JBIC J. Boil. Inorg. Chem..

[28] Novinec M., Kordiš D., Turk V., Lenarčič B. (2005). Diversity and Evolution of the Thyroglobulin Type-1 Domain Superfamily. Mol. Boil. Evol..

[29] Meh P., Pavšič M., Turk V., Baici A., Lenarčič B. (2005). Dual concentration-dependent activity of thyroglobulin type-1 domain of testican: Specific inhibitor and substrate of cathepsin L.. Boil. Chem..

[30] Wu C.-J., Feng X., Lu M., Morimura S., Udey M.C. (2017). Matriptase-mediated cleavage of EpCAM destabilizes claudins and dysregulates intestinal epithelial homeostasis. J. Clin. Investig..

[31] Trebak M., Begg G.E., Chong J.M., Kanazireva E.V., Herlyn D., Speicher D.W. (2000). Oligomeric State of the Colon Carcinoma-associated Glycoprotein GA733-2 (Ep-CAM/EGP40) and Its Role in GA733-mediated Homotypic Cell-Cell Adhesion. J. Boil. Chem..

[32] Münz M. (2008). Glycosylation is crucial for stability of tumour and cancer stem cell antigen EpCAM. Front. Biosci..

[33] Pavšič M., Ilc G., Vidmar T., Plavec J., Lenarčič B. (2015). The cytosolic tail of the tumor marker protein Trop2 - a structural switch triggered by phosphorylation. Sci. Rep..

[34] Casaletto J.B., Geddie M.L., Abu-Yousif A.O., Masson K., Fulgham A., Boudot A., Maiwald T., Kearns J.D., Kohli N., Su S. (2019). MM-131, a bispecific anti-Met/EpCAM mAb, inhibits HGF-dependent and HGF-independent Met signaling through concurrent binding to EpCAM. Proc. Natl. Acad. Sci. USA.

[35] Jiang L., Zhang C., Li Y., Yu X., Zheng J., Zou P., Li Y., Bin X., Lu J., Zhou Y. (2010). A non-synonymous polymorphism Thr115Met in the EpCAM gene is associated with an increased risk of breast cancer in Chinese population. Breast Cancer Res. Treat..

[36] Hall B.M., Roberts S.A., Heroux A., Cordes M.H.J. (2008). Two structures of a lambda Cro variant highlight dimer flexibility but disfavor major dimer distortions upon specific binding of cognate DNA. J. Mol. Biol..

[37] Ahlstrom L.S., Miyashita O. (2013). Packing Interface Energetics in Different Crystal Forms of the λ Cro Dimer. Proteins Struct. Funct. Bioinform..

[38] Litvinov S.V., Bakker H.A.M., Gourevitch M.M., Velders M.P., Warnaar S.O. (1994). Evidence for a Role of the Epithelial Glycoprotein 40 (Ep-CAM) in Epithelial Cell-Cell Adhesion. Cell Adhes. Commun..

[39] Balzar M., Bruijn I.H.B.-D., Rees-Bakker H.A.M., Prins F.A., Helfrich W., De Leij L., Riethmüller G., Alberti S., Warnaar S.O., Fleuren G.J. (2001). Epidermal Growth Factor-Like Repeats Mediate Lateral and Reciprocal Interactions of Ep-CAM Molecules in Homophilic Adhesions. Mol. Cell. Boil..

[40] Levy E.D., Erba E., Robinson C.V., Teichmann S.A. (2008). Assembly reflects evolution of protein complexes. Nat..

[41] Balzar M., Prins F.A., Bakker H.A., Fleuren G.J., Warnaar S.O., Litvinov S.V. (1999). The Structural Analysis of Adhesions Mediated by Ep-CAM. Exp. Cell Res..

[42] Fornaro M., Arciprete R.D., Stella M., Bucci C., Nutini M., Capri M.G., Alberti S. (1995). Cloning of the gene encoding TROP-2, a cell-surface glycoprotein expressed by human carcinomas. Int. J. Cancer.

[43] Guillemot J.-C., Naspetti M., Malergue F., Montcourrier P., Galland F., Naquet P. (2001). Ep-CAM transfection in thymic epithelial cell lines triggers the formation of dynamic actin-rich protrusions involved in the organization of epithelial cell layers. Histochem. Cell Boil..

[44] Litvinov S.V., Balzar M., Winter M.J., Bakker H.A., Bruijn I.H.B.-D., Prins F., Fleuren G.J., Warnaar S.O. (1997). Epithelial Cell Adhesion Molecule (Ep-CAM) Modulates Cell–Cell Interactions Mediated by Classic Cadherins. J. Cell Boil..

[45] Winter M.J., Nagelkerken B., E E Mertens A., Rees-Bakker H.A.M., Bruijn I.H.B.-D., Litvinov S.V. (2003). Expression of Ep-CAM shifts the state of cadherin-mediated adhesions from strong to weak. Exp. Cell Res..

[46] Ladwein M., Pape U.-F., Schmidt D.-S., Schnölzer M., Fiedler S., Langbein L., Franke W., Moldenhauer G., Zöller M. (2005). The cell–cell adhesion molecule EpCAM interacts directly with the tight junction protein claudin-7. Exp. Cell Res..

[47] Wu C.-J., Mannan P., Lu M., Udey M.C. (2013). Epithelial Cell Adhesion Molecule (EpCAM) Regulates Claudin Dynamics and Tight Junctions. J. Boil. Chem..

[48] Salomon J., Gaston C., Magescas J., Duvauchelle B., Canioni D., Sengmanivong L., Mayeux A., Michaux G., Campeotto F., Lemale J. (2017). Contractile forces at tricellular contacts modulate epithelial organization and monolayer integrity. Nat. Commun..

[49] Maghzal N., Vogt E., Reintsch W., Fraser J.S., Fagotto F. (2010). The tumor-associated EpCAM regulates morphogenetic movements through intracellular signaling. J. Cell Boil..

[50] Maghzal N., Kayali H.A., Rohani N., Kajava A., Fagotto F. (2013). EpCAM Controls Actomyosin Contractility and Cell Adhesion by Direct Inhibition of PKC. Dev. Cell.

[51] Hsu Y.-T., Osmulski P., Wang Y., Huang Y.-W., Liu L., Ruan J., Jin V.X., Kirma N.B., Gaczyńska M., Huang T.H.-M. (2016). EpCAM-Regulated Transcription Exerts Influences on Nanomechanical Properties of Endometrial Cancer Cells That Promote Epithelial-to-Mesenchymal Transition. Cancer Res..

[52] Hachmeister M., Bobowski K.D., Hogl S., Dislich B., Fukumori A., Eggert C., Mack B., Kremling H., Sarrach S., Coscia F. (2013). Regulated Intramembrane Proteolysis and Degradation of Murine Epithelial Cell Adhesion Molecule mEpCAM. PLOS ONE.

[53] Song Y., DiMaio F., Wang R.Y.-R., Kim D., Miles C., Brunette T., Thompson J., Baker D. (2013). High-resolution comparative modeling with RosettaCM. Struct..

[54] Raman S., Vernon R., Thompson J., Tyka M., Sadreyev R., Pei J., Kim D., Kellogg E., DiMaio F., Lange O. (2009). Structure prediction for CASP8 with all-atom refinement using Rosetta. Proteins Struct. Funct. Bioinform..

[55] Lichtenthaler S.F., Haass C., Steiner H. (2011). Regulated intramembrane proteolysis - lessons from amyloid precursor protein processing. J. Neurochem..

[56] Münz M., Kieu C., Mack B., Schmitt B., Zeidler R., Gires O., M M. (2004). The carcinoma-associated antigen EpCAM upregulates c-myc and induces cell proliferation. Oncogene.

[57] Chaves-Pérez A., Mack B., Maetzel D., Kremling H., Eggert C., Harréus U., Gires O. (2012). EpCAM regulates cell cycle progression via control of cyclin D1 expression. Oncogene.

[58] Huang Y., Chanou A., Kranz G., Pan M., Kohlbauer V., Ettinger A., Gires O. (2018). Membrane-associated epithelial cell adhesion molecule is slowly cleaved by γ-secretase prior to efficient proteasomal degradation of its intracellular domain. J. Boil. Chem..

[59] Dejanović L. (2017). Design and Preparation of Various Forms of EpCAM’s Intracellular Domain. BSc Thesis.

[60] Tretter J.Y. (2017). Innovative Therapy Modalities for Solid EpCAM-Positive Tumors. Ph.D. Thesis.

[61] Martin B., Schneider R., Janetzky S., Waibler Z., Pandur P., Kühl M., Behrens J., Von Der Mark K., Starzinski-Powitz A., Wixler V. (2002). The LIM-only protein FHL2 interacts with β-catenin and promotes differentiation of mouse myoblasts. J. Cell Boil..

[62] Sun J., Weis W.I. (2010). Biochemical and Structural Characterization of β-Catenin Interactions with Nonphosphorylated and CK2-Phosphorylated Lef-1. J. Mol. Boil..

[63] Love J.J., Li X., Case D.A., Giese K., Grosschedl R., Wright P.E., Crosschedl R. (1995). Structural basis for DNA bending by the architectural transcription factor LEF-1. Nature.

[64] Russel D., Lasker K., Webb B., Velázquez-Muriel J., Tjioe E., Schneidman-Duhovny D., Peterson B., Sali A. (2012). Putting the pieces together: Integrative modeling platform software for structure determination of macromolecular assemblies. PLoS Boil..

[65] Valenta T., Hausmann G., Basler K. (2012). The many faces and functions of β-catenin. EMBO J..

[66] Fernandez M.A., Biette K.M., Dolios G., Seth D., Wang R., Wolfe M.S. (2016). Transmembrane Substrate Determinants for γ-Secretase Processing of APP CTFβ. Biochemistry.

[67] Van Zundert G., Rodrigues J., Trellet M., Schmitz C., Kastritis P., Karaca E., Melquiond A., Van Dijk M., De Vries S., Bonvin A. (2016). The HADDOCK2.2 Web Server: User-Friendly Integrative Modeling of Biomolecular Complexes. J. Mol. Boil..

[68] Koprowski H., Steplewski Z., Mitchell K., Herlyn M., Herlyn R., Fuhrer P. (1979). Colorectal carcinoma antigens detected by hybridoma antibodies. Somat. Cell Mol. Genet..

[69] Herlyn R., Herlyn M., Steplewski Z., Koprowski H. (1979). Monoclonal antibodies in cell-mediated cytotoxicity against human melanoma and colorectal carcinoma. Eur. J. Immunol..

[70] Göitlinger H.G., Funke I., Johnson J.P., Gokel J.M., Riethmüller G. (1986). The epithelial cell surface antigen 17–1A, a target for antibody-mediated tumor therapy: Its biochemical nature, tissue distribution and recognition by different monoclonal antibodies. Int. J. Cancer.

[71] Simon M., Stefan N., Plückthun A., Zangemeister-Wittke U. (2013). Epithelial cell adhesion molecule-targeted drug delivery for cancer therapy. Expert Opin. Drug Deliv..

[72] Spizzo G., Obrist P., Ensinger C., Theurl I., Dünser M., Ramoni A., Gunsilius E., Eibl G., Mikuz G., Gastl G. (2002). Prognostic significance of Ep-CAM AND Her-2/neu overexpression in invasive breast cancer. Int. J. Cancer.

[73] Spizzo G., Gastl G., Obrist P., Went P., Dirnhofer S., Bischoff S., Mirlacher M., Sauter G., Simon R., Stopatschinskaya S. (2004). High Ep-CAM Expression is Associated with Poor Prognosis in Node-positive Breast Cancer. Breast Cancer Res. Treat..

[74] Varga M., Obrist P., Schneeberger S., Mühlmann G., Felgel-Farnholz C., Fong D., Zitt M., Brunhuber T., Schäfer G., Gastl G. (2004). Overexpression of epithelial cell adhesion molecule antigen in gallbladder carcinoma is an independent marker for poor survival. Clin. Cancer Res..

[75] Fong D., Steurer M., Obrist P., Barbieri V., Margreiter R., Amberger A., Laimer K., Gastl G., Tzankov A., Spizzo G. (2007). Ep-CAM expression in pancreatic and ampullary carcinomas: Frequency and prognostic relevance. J. Clin. Pathol..

[76] Brunner A., Schaefer G., Veits L., Brunner B., Prelog M., Ensinger C. (2008). EpCAM overexpression is associated with high-grade urothelial carcinoma in the renal pelvis. Anticancer. Res..

[77] Scheunemann P., Stoecklein N.H., Rehders A., Bidde M., Metz S., Peiper M., Eisenberger C.F., Esch J.S.A., Knoefel W.T., Hosch S.B. (2007). Occult tumor cells in lymph nodes as a predictor for tumor relapse in pancreatic adenocarcinoma. Langenbecks Archiv. für Chirurgie.

[78] Chen X., Ma W.-Y., Xu S.-C., Liang Y., Fu Y.-B., Pang B., Xin T., Fan H.-T., Zhang R., Luo J.-G. (2014). The overexpression of Epithelial cell adhesion molecule (EpCAM) in glioma. J. Neuro-Oncol..

[79] Trzpis M., McLaughlin P.M., De Leij L.M., Harmsen M.C. (2007). Epithelial Cell Adhesion Molecule. Am. J. Pathol..

[80] Ligtenberg M.J., Kuiper R.P., Chan T.L., Goossens M., Hebeda K.M., Voorendt M., Lee T.Y.H., Bodmer D., Hoenselaar E., Hendriks-Cornelissen S.J.B. (2008). Heritable somatic methylation and inactivation of MSH2 in families with Lynch syndrome due to deletion of the 3′ exons of TACSTD_1_. Nat. Genet..

[81] Kovacs M.E., Papp J., Szentirmay Z., Ottó S., Olah E. (2009). Deletions removing the last exon ofTACSTD1constitute a distinct class of mutations predisposing to Lynch syndrome. Hum. Mutat..

[82] Kuiper R.P., Vissers L.E.L.M., Venkatachalam R., Bodmer D., Hoenselaar E., Goossens M., Haufe A., Kamping E., Niessen R.C., Hogervorst F.B. (2011). Recurrence and variability of germline EPCAM deletions in Lynch syndrome. Hum. Mutat..

[83] Pathak S.J., Mueller J.L., Okamoto K., Das B., Hertecant J., Greenhalgh L., Cole T., Pinsk V., Yerushalmi B., Gurkan O.E. (2018). EPCAM mutation update: Variants associated with congenital tufting enteropathy and Lynch syndrome. Hum. Mutat..

[84] Reifen R.M., Cutz E., Griffiths A.M., Ngan B.Y., Sherman P.M. (1994). Tufting enteropathy: A newly recognized clinicopathological entity associated with refractory diarrhea in infants. J. Pediatr. Gastroenterol. Nutr..

[85] Sivagnanam M., Mueller J.L., Lee H., Chen Z., Nelson S.F., Turner D., Zlotkin S.H., Pencharz P.B., Ngan B., Libiger O. (2008). Identification of EpCAM as the Gene for Congenital Tufting Enteropathy. Gastroenterology.

[86] Schnell U., Kuipers J., Mueller J.L., Veenstra-Algra A., Sivagnanam M., Giepmans B.N.G. (2013). Absence of cell-surface EpCAM in congenital tufting enteropathy. Hum. Mol. Genet..

[87] Al-Mayouf S.M., Alswaied N., Alkuraya F.S., AlMehaidib A., Faqih M. (2009). Tufting Enteropathy and Chronic Arthritis: A Newly Recognized Association With a Novel EpCAM Gene Mutation. J. Pediatr. Gastroenterol. Nutr..

[88] Salomon J., Espinosa-Parrilla Y., Goulet O., Al-Qabandi W., Guigue P., Canioni D., Bruneau J., Alzahrani F., Almuhsen S., Cerf-Bensussan N. (2011). A founder effect at the EPCAM locus in Congenital Tufting Enteropathy in the Arabic Gulf. Eur. J. Med. Genet..

[89] Sivagnanam M., Schaible T., Szigeti R., Byrd R.H., Finegold M.J., Ranganathan S., Gopalakrishna G., Tatevian N., Kellermayer R. (2010). Further evidence for EpCAM as the gene for congenital tufting enteropathy. Am. J. Med. Genet. Part. A.

[90] Thoeni C., Amir A., Guo C., Zhang S., Avitzur Y., Heng Y., Cutz E., Muise A.M. (2014). A Novel Nonsense Mutation in the EpCAM Gene in a Patient With Congenital Tufting Enteropathy. J. Pediatr. Gastroenterol. Nutr..

[91] Salomon J., Goulet O., Canioni D., Brousse N., Lemale J., Tounian P., Coulomb A., Marinier E., Hugot J., Ruemmele F. (2013). Genetic characterization of congenital tufting enteropathy: Epcam associated phenotype and involvement of SPINT2 in the syndromic form. Qual. Life Res..

[92] D’Apolito M., Pisanelli D., Faletra F., Giardino I., Gigante M., Pettoello-Mantovani M., Goulet O., Gasparini P., Campanozzi A. (2015). Genetic analysis of Italian patients with congenital tufting enteropathy. World J. Pediatr..

[93] Tan Q.K., Cardona D.M., Rehder C.W., McDonald M. (2017). Identification of EPCAM mutation: Clinical use of microarray. Clin. Case Rep..

[94] Tang W., Huang T., Xu Z., Huang Y. (2018). Novel Mutations in EPCAM Cause Congenital Tufting Enteropathy. J. Clin. Gastroenterol..

[95] Fang Y., Luo Y., Yu J., Chen J. (2020). A case of severe malnutrition infant with neonatal onset intractable diarrhea. BMC Pediatr..

[96] Das B., Okamoto K., Rabalais J., Marchelletta R., Barrett K., Das S., Niwa M., Sivagnanam M. (2020). Congenital Tufting Enteropathy-Associated Mutant of Epithelial Cell Adhesion Molecule Activates the Unfolded Protein Response in a Murine Model of the Disease. Cells.

[97] Nagao K., Zhu J., Heneghan M.B., Hanson J.C., Morasso M.I., Tessarollo L., Mackem S., Udey M.C. (2009). Abnormal Placental Development and Early Embryonic Lethality in EpCAM-Null Mice. PLoS ONE.

[98] Guerra E., Lattanzio R., La Sorda R., Dini F., Tiboni G.M., Piantelli M., Alberti S. (2012). mTrop1/Epcam knockout mice develop congenital tufting enteropathy through dysregulation of intestinal E-cadherin/β-catenin. PLoS ONE.

[99] Mueller J.L., McGeough M.D., Pena C.A., Sivagnanam M. (2013). Functional consequences of EpCam mutation in mice and men. Am. J. Physiol. Liver Physiol..

[100] Kozan P.A., McGeough M.D., Pena C.A., Mueller J.L., Barrett K.E., Marchelletta R.R., Sivagnanam M. (2014). Mutation of EpCAM leads to intestinal barrier and ion transport dysfunction. J. Mol. Med..

[101] Nübel T., Preobraschenski J., Tuncay H., Weiss T., Kuhn S., Ladwein M., Langbein L., Zöller M. (2009). Claudin-7 Regulates EpCAM-Mediated Functions in Tumor Progression. Mol. Cancer Res..

